# Neo‐Antigen‐Reactive T Cells Immunotherapy for Colorectal Cancer: A More Personalized Cancer Therapy Approach

**DOI:** 10.1002/gch2.202200186

**Published:** 2023-10-17

**Authors:** Guan‐Liang Chen, De‐Xia Kong, Yan Lin

**Affiliations:** ^1^ Department of Gastroenterology Surgery Affiliated Hospital of Shaoxing University Shaoxing 312000 China; ^2^ Center for General Practice Medicine Department of Gastroenterology Zhejiang Provincial People's Hospital (Affiliated People's Hospital), Hangzhou Medical College No. 158 Shangtang Road Hangzhou Zhejiang 310014 China

**Keywords:** colorectal cancer, neoantigen, neoantigen‐reactive T cell

## Abstract

Colorectal cancer (CRC) is the second most common malignancy in women and the third most frequent cancer in men. Evidence has revealed that the survival of patients with metastatic CRC is very low, between one and three years. Neoantigens are known proteins encoded by mutations in tumor cells. It is theorized that recognizing neoantigens by T cells leads to T cell activation and further antitumor responses. Neoantigen‐reactive T cells (NRTs) are designed against the mentioned neoantigens expressed by tumor cells. NRTs selectively kill tumor cells without damage to non‐cancerous cells. Identifying patient‐specific and high immunogen neoantigens is important in NRT immunotherapy of patients with CRC. However, the main challenges are the side effects and preparation of NRTs, as well as the effectiveness of these cells in vivo. This review summarized the properties of neoantigens as well as the preparation and therapeutic outcomes of NRTs for the treatment of CRC.

## Graphical abstract

Neoantigen‐reactive T cells (NRTs) are designed against the neoantigens expressed by tumor cells. NRTs selectively kill tumor cells without damage to non‐cancerous cells. Identifying patient‐specific and high immunogen neoantigens is important in NRT immunotherapy of patients with colorectal cancer. This review summarized the properties of neoantigens as well as the preparation and therapeutic outcomes of NRTs for the treatment of colorectal cancer.

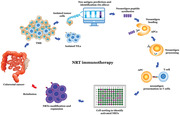

## 1 Introduction

According to available statistics, colorectal cancer (CRC) is the third deadliest cancer globally, affecting about two million people annually.^[^ ^1^ ^]^ In 2021, 104270 new cases of colon cancer and 45230 new cases of rectal cancer were reported in the United States.^[^ ^2^ ^]^ CRC is most common in people over 50 in developing countries. Evidence suggests that several factors, including obesity, smoking, Western diets, high red meat consumption, alcohol consumption, and inactivity, can increase the risk of developing CRC.^[^ ^3^ ^]^ In CRC treatment, surgery is a common method; however, adjuvant chemotherapy is also used in grade II and III CRC.^[^ ^4^, ^5^ ^]^ These therapeutic methods face several limitations and challenges. For instance, in surgery, the possibility of cancer recurrence is very high due to incomplete tumor resection. Chemotherapy also has various side effects; sometimes, tumor cells become resistant to it.^[^ ^6^, ^7^ ^]^ Recently, immunotherapeutic agents have been used for all advanced microsatellite instability‐high (MSI‐H): DNA mismatch repair–deficient (dMMR) solid tumors, such as metastatic CRC, resulting in durable clinical outcomes and improved survival rates in treated patients. Regrettably, more than 90% of patients with CRC are not MSI‐H: dMMR, and methods based on immunotherapy have not had much effect on this group of patients.^[^ ^8^ ^]^ Several clinical trials using checkpoint inhibitors such as pembrolizumab and nivolumab (anti‐programmed death 1 [PD‐1] antibodies), as well as the combination of nivolumab with ipilimumab (cytotoxic T‐lymphocyte‐associated protein 4 [CTLA‐4]), have been performed in MSI‐H metastatic CRC. The results of these studies have been relatively satisfactory.^[^ ^9^, ^10^, ^11^ ^]^ Despite the increase in the survival of CRC patients through the implementation of screening programs and targeted therapy in recent decades, the relative five‐year survival of CRC patients is still only ≈68%.^[^ ^12^ ^]^

Evidence demonstrated that MSI‐H CRC is associated with increased frequency of tumor‐infiltrating lymphocytes (TILs) and high rates of tumor mutation, which justifies the use of various T cell‐associated immunotherapies, such as using immune checkpoint inhibitors, neoantigen‐reactive T cell (NRT) therapy and chimeric antigen receptor (CAR)‐T cells in MSI‐H CRC.^[^ ^13^ ^]^ However, immunotherapy for CRC treatment still has a long way to go because immune checkpoint blockers’ effectiveness and success rate have been very low.^[^ ^14^ ^]^ Another method of immunotherapy is identifying and using neoantigens. The emergence of novel techniques, such as next‐generation sequencing (NGS), helps identify high‐immunogen neoantigens to produce personalized medicine neoantigen‐based vaccines or NRTs.^[^ ^15^ ^]^ This cancer vaccine has been examined in various human cancers such as melanoma, CRC, glioblastoma, and other malignancies; however, the clinical outcomes of these cancer vaccines were not satisfactory.^[^ ^16^, ^17^ ^]^ In CRC, it has been reported that immunotherapy using human leukocyte antigen (HLA)‐C*08:02‐restricted TILs targeting mutated Kirsten rat sarcoma virus (KRAS)^G12D^ oncogene could inhibit tumor progression.^[^ ^18^ ^]^

Recently it has been reported that the adoptive NRTs transferring induced by cancer vaccines containing mutant peptides effectively repressed tumor cell growth in CRC‐bearing mice models.^[^ ^19^ ^]^ Moreover, the coexpression of CD39 and CD103 is a hallmark of neoantigen‐specific CD8^+^ T cells in patients with MMR‐proficient CRC and low mutation burden. Consequently, identifying these subsets for engineering TCRs and adoptive T cell transfer might be a potential therapeutic approach to treat patients with CRC.^[^ ^20^ ^]^ Although the efficacy and side effects of NRT immunotherapy have not yet been well studied due to its high specificity against antigens expressed by tumor cells, this therapeutic approach could be more successful in treating cancer in the future. In this regard, it has been revealed that neoantigen‐specific antitumor immune response was identified in more than half of the vaccinated microsatellite stability (MSS) CRC patients with postoperative recurrence or metastasis.

Additionally, progression‐free survival (PFS) was significantly prolonged in the patients (19 months). These findings indicate that personalized neoantigen vaccine therapy could be effective, feasible, and safe in MSS CRC patients.^[^ ^21^ ^]^ Therefore, according to the limitations and challenges of other treatments in CRC, it seems that NRT therapy can achieve satisfactory clinical outcomes for patients with CRC. This review summarized the properties of neoantigens and the role of NRTs in CRC treatment, along with the challenges and opportunities ahead.

## 2 Neoantigens

Tumor‐specific peptides are known as neoantigens presented via MHCs‐I on the surface of cancer cells, initiating a T cell‐mediated cytotoxic antitumor immune response and expanding specific T cells.^[^ ^22^, ^23^ ^]^ Tumor cells bearing mutations produce these neoantigens, affecting protein sequence and including codon insertion, codon deletion, nonsynonymous point mutations, splicing mutations, gene fusions, and frameshift mutations.^[^ ^24^ ^]^ Some neoantigens have high specificity and immunogenicity and may be potential targets for immunotherapy.^[^ ^25^ ^]^ Scientists hypothesized in the 1980s and 1990s that tumor‐specific antigens (TSAs) such as neoantigens could be presented on the surface of tumor cells by HLA class I to recognize by effector T cells providing anti‐tumor responses and tumor cells elimination.^[^ ^26^ ^]^ Due to the high cost of traditional methods for identifying the mutated genes and tumor‐derived neoantigens, it is easier and more economical to use whole‐genome sequencing (WGS) and whole‐exon sequencing (WES) methods.

On the other hand, recognizing neoantigens can lead to identifying their specific T cells, which can help develop immunotherapy‐based treatments.^[^ ^27^, ^28^, ^29^ ^]^ Neoantigens belong to a group of TSAs and are different from tumor‐associated antigens (TAAs) because, unlike TTAs, they are not expressed by non‐tumor cells, are not affected by immune system tolerance mechanisms, have higher immunogenicity and affinity for binding to the MHC and TCR molecules.^[^ ^30^ ^]^ Based on the available knowledge, the degree of abnormal protein alienation rises with increasing differences between the mutant sequence and the encoding gene's main sequence, leading to increased immunogenicity. Point mutations are usually involved in the formation of most of these proteins. However, frame‐shift and insertion‐deletions (indels) mutations can also be involved in this process, and the effect of these two mutations on the amino acid sequence and protein conformation has a higher affinity for binding to the MHCs and increase the probability of being identified as neoantigens by T cells.^[^ ^31^, ^32^, ^33^ ^]^ However, only up to two percent of T cells naturally and spontaneously identify and respond to these neoantigens, which cannot be efficient as an anti‐tumor response. As a result, empowering autologous NRTs in vitro and returning them to the body can increase the success of cancer immunotherapy.^[^ ^33^, ^34^ ^]^ Various neoantigens have been identified based on the latest studies on human cancers, and analyses are underway. The most important of which are murine sarcoma viral oncogene homolog B1 (BRAF), KRAS, TP53, HRAS/KRAS/ NRAS, and the characteristics of the mentioned neoantigens are shown in **Table** **1**.^[^ ^35^, ^36^, ^37^ ^]^ However, there are also patient‐specific neoantigens that can be predicted by bioinformatics software such as HLAMiner and Polysolver.^[^ ^38^ ^]^ For instance, TSHZ3‐L523P, RARA‐R83H, TP53‐R248W, EYA2‐V333I, and NRAS‐G12D specific‐patient neoantigens were predicted to induce NRT antitumor responses in CRC.^[^ ^19^ ^]^ Although neoantigens are potential targets for personalized vaccines and NRT therapy, most neoantigen‐targeted therapies are customized and costly. To overcome these limitations, “shared neoantigens” could be more suitable for patients. Shared neoantigens are identified via available neoantigen prediction algorithms, providing a valuable target list for off‐the‐shelf immunotherapies.^[^ ^39^ ^]^

**Table 1:** The most important neoantigens in human cancers.

Gene	Protien modification	HLA	Top cancer	Mutation frequency	Ref
BRAF	p.Val600Glu	A*02	Melanoma	43.9	[114]
BRAF	p.Val600Glu	B*27:05	Melanoma	43.9	[115]
KRAS	p.Gly12Asp	A*03	Pancreas	32.4	[116]
KRAS	p.Gly12Asp	A*11:01	Pancreas	32.4	[77]
KRAS	p.Gly12Asp	C*08:02	Pancreas	32.4	[117]
HRAS/KRAS/ NRAS	p.Gln61Arg	A*01:01	Melanoma	12.4	[116, 118]
KRAS	p.Gly12Val	A*03:01	Lung	6.7	[119]
KRAS	p.Gly12Va	B*35	Lung	6.7	[120]
KRAS	p.Gly12Val	A*11:01	Lung	6.7	[121]
TP53	p.Arg175His	A*02:01	Colorectum	6.5	[121, 122, 123, 124]

## 3 Neoantigen‐Reactive T Cells

Because of the ability of NRTs to detect and eliminate tumor cells by detecting and responding to tumor‐expressed neoantigens without damage to normal cells, they can overcome several of the challenges of cancer therapy. However, the most challenging part of developing NRT immunotherapy is identifying and expanding NRTs in vitro.^[^ ^36^ ^]^ In NRT immunotherapy, CD8^+^ TILs and tumor cells are isolated from patients with cancer. However, studies are ongoing to develop protocols for identifying NRTs from healthy donor T cells.^[^ ^40^ ^]^ Sequencing is employed to identify the neoantigens expressed by tumor cells. After this step, CD8^+^ T cells are co‐cultured with neoantigen‐presenting cells to sort and isolate tumor‐specific NRTs. After in vitro modification and expansion, NRTs are ready to be reinfused into the patient's body.^[^ ^41^ ^]^ In this section, the basic procedures of NRT immunotherapy are briefly discussed.

### 3.1 Prediction of Putative Neoantigens and Verification

Effective identifying neoantigens with high immunogenicity by traditional genomic methods needs combining neoantigen prediction and prioritization. Prediction of neoantigens necessitates sample acquisition, high‐quality sequencing data, predicting the somatic mutations present in the cancer cell, and predicting the somatic mutations‐derived neoantigens.^[^ ^42^ ^]^ The primary phase in NRT immunotherapy is to find and select the putative neoantigen. Comparing normal and tumor cell RNA or DNA sequences leads to detect nonsynonymous variants. Prediction of the binding affinity of MHC‐I proteins and their peptide ligands plays a critical role in vaccine design and NRT immunotherapy. The input of sequences of variants could predict putative neoantigens into the machine learning models, such as MHCflurry, NetMHC, and NetMHCpan. These are the most important machine‐learning models that use algorithms to predict the MHC binding affinity.^[^ ^43^, ^44^, ^45^ ^]^ Nonetheless, the *in silico* neoantigen prediction algorithms output could have a relatively high false‐positive outcome.^[^ ^46^ ^]^ Therefore, algorithms can be modified to improve the specificity of neoantigen prediction. MHCflurry uses a new architecture and peptide encoding scheme, and its 1.2.0 version utilizes datasets of mass spectrometry for selecting models. A small benchmark of affinity measurements revealed that MHCflurry 1.2.0 displays high accuracy when compared with NetMHCpan 4.0. Moreover, the prediction speed in MHCflurry is more than 7000 predictions/second, ≈400 times faster than NetMHCpan 4.0.^[^ ^43^ ^]^ NetMHC is based on the “allele‐specific” method. It means distinct predictors are proficient for each MHC allele, and the model's input is the peptide of interest.^[^ ^47^ ^]^ In contrast, NetMHCpan utilizes a “pan‐allele” method in which a single model takes both the peptide and an MHC allele representation as input.^[^ ^48^ ^]^ With two‐output neural networks, NetMHCpan 4.0 generates predictions for binding affinity and the likelihood of mass spectrometry identification using peptides eluted from MHC and identified by mass spectrometry in its training set.^[^ ^44^ ^]^ In CRC, these methods can be used for comparing immunopeptidomics outcomes. For instance, an investigation reported that NetMHCpan could only predict 8‐mer from mediator complex subunit 25 (*MED25*) and 10‐mer from flavin‐containing dimethylaniline monoxygenase 5 (*FMO5*) genes as strong binders identified by mass spectrometry.^[^ ^49^ ^]^

Another study on CRC employed several software, including NetMHC, NetMHCpan, PickPocket, PSSMHCpan, and SMM, to predict affinity between HLA alleles and neoantigen peptides. Following the prediction of neoantigens, EPIC software was used to predict candidate high‐affinity peptides. EPIC predicts neoantigens regarding the mass spectrometry‐derived motifs and the expression of tissue‐specific profiles. Accordingly, EPIC software uses various complex factors in antigen presentation, including affinity and the expression of tumor‐specific genes, predicting epitope presentation more accurately.^[^ ^50^ ^]^

Despite the advantages mentioned about these methods, their predictive immunogenicity accuracy is low. To overcome this challenge, TruNeo, as an integrated computational pipeline, is designed for identifying and selecting highly immunogenic neoantigens. The prediction of this method is based on several biological processes, which is faster and more accurate than only predicting peptide‐MHC binding affinity methods. Consequently, TruNeo can provide candidate immunogenic neoantigens prioritization for neoantigen‐based personalized therapies.^[^ ^51^ ^]^

On the other hand, mass spectrometry data are more accurate.^[^ ^52^, ^53^ ^]^ However, mass spectrometry needs more samples compared to NGS. Another challenge with mass spectrometry is ignoring some neo‐peptides expressed on the cancer cell's surface.

Following neoantigen prediction, synthesized tandem minigene (TMG) and long peptides are pulsed or transfected into neoantigen‐presenting cells to present neoantigens.^[^ ^54^ ^]^ Next, neoantigen‐presenting cells are loaded by special reporters using a T‐scan platform.^[^ ^55^ ^]^ Enzymes released by T cells, such as granzyme B, can cleave loaded reporters, leading to fluorescence excitation.^[^ ^56^ ^]^ Using biotin to label cell membrane proteins of T cells is another method of NRT preparation. Applying N‐hydroxysuccinimide‐biotin to label cell membrane proteins on T cells' surface is another NRT preparation method. This method is based on the trogocytosis phenomena and the transfer of N‐hydroxysuccinimide‐biotin‐labeled T cell surface proteins to the surface of T‐cell‐specific neoantigen presenting cells. In this way, these cells can be identified and tracked by fluorescence‐activated cell sorting (FACS).^[^ ^57^, ^58^ ^]^ Moreover, in neoantigen‐presenting cells, signaling and antigen‐presenting bifunctional receptors (SABRs) containing stimulatory domains can effectively induce TCR‐like signals following interaction with antigen‐specific T cells.

The alternative technique directly presenting neoantigens is peptide‐MHC (pMHC) tetramers labeled with fluorescein. Following the recognition of pMHC tetramers by specific T cells, FACS is used to sort the neoantigen‐specific T cells.^[^ ^59^ ^]^  In contrast to the TMGs mentioned earlier and long peptides, this method does not require autologous neoantigen‐presenting cells, which is one of the advantages of using pMHC tetramers. Furthermore, pMHC tetramers can present every neoantigen similarly and effectively.^[^ ^60^ ^]^ However, in addition to the advantages of this method, there are weaknesses. For example, since pMHCs cannot process antigens naturally like APCs and lack co‐stimulatory molecules, they may not effectively present the loaded neoantigens to T cells. Furthermore, unoptimized pMHC staining protocols, the high complexity of the method, and sorting complications are other limitations of using pMHCs. It has also been reported that direct read‐out of neoantigen sequences identified by T cells is impossible.^[^ ^36^, ^61^ ^]^

### 3.2 Selection of NRTs

Studies show two ways to identify specific neoantigen‐specific cells, including using pMHC tetramer and the other Abeling Immune Partnerships by SorTagging Intercellular Contacts (LIPSTIC).^[^ ^62^ ^]^ A fundamental problem with pMHC tetramer is the lack of confirmation of activated T cells following antigen recognition. Sometimes T cell binds to the antigen only through TCR without initiating downstream signals and T cell activation. To overcome this problem, researchers use a robotic microscope system to record the mechanical force between the TCR and the antigens, showing the effective binding of T cells to the antigen and confirming its activation. The LIPSTIC method is based on transferring an intracellular substrate that detects T cells and APC interaction and uses a cytokine indicator of activated T cells such as interferon‐gamma (IFN‐γ). However, this indicator is inappropriate because IFN‐γ is multisource, so tracking the single producer cell is difficult. Therefore, LIPSTIC is more applicable for determining the frequency of activated T cell clusters by neoantigens.^[^ ^54^, ^63^ ^]^

On the other hand, specific cell markers by flow cytometry are always improved options for cell identification, and these markers can also be used for activated T cells. In the case of TILs, it has been shown that PD‐1, lymphocyte activating 3 (LAG‐3), T cell immunoglobulin, and mucin‐domain containing‐3 (Tim‐3), 4‐1BB, CD39, and CD103 markers can be suitable candidates, but in the identification of NRTs, the results of studies indicate that 4‐1BB is a better marker.^[^ ^64^, ^65^ ^]^ Biosensors can also be designed to detect T cell activation, such as increased intracellular calcium levels, enzymes involved in signaling pathways such as zap70, and transcription factors such as the nuclear factor of activated T‐cells (NFAT) to help identify activated T cells. These sensors include the ROZA FRET sensor, which detects activities of ZAP 70 kinase; the LIBRA system, which contains an IP3 binding domain between CFP and YFP; Ca2^+^ sensors that bind to intramolecular Ca2^+^ and transform its conformation and change FRET signal, and NFAT reporters fused with a YFP that retain nuclear localization domain but not the DNA‐binding domain.^[^ ^66^, ^67^, ^68^, ^69^, ^70^, ^71^ ^]^

### 3.3 Optimization of NRTs In Vivo

Due to the challenges and limitations of immunotherapy with NRTs, such as low persistence and loss of T cells performance, limitations of tumor tissue penetration and ineffective trafficking, as well as immunosuppressive TME, strengthening NRTs and also trying to overcome these complications effectively is necessary for the success of the NRT immunotherapy.^[^ ^72^ ^]^ Researchers use different strategies to overcome these limitations in NRT immunotherapy. For example, employing cytokines, co‐stimulatory molecules, and growth factors improve the expansion and persistence of NRTs. Furthermore, removing physical barriers, inhibiting oncogenic signaling pathways, regulatory T cells (Tregs) depletion, and use of drug‐receptor delivery signals, checkpoint blockers, and nanoparticles in drug delivery could increase the effectiveness of NRT immunotherapy.^[^ ^36^ ^]^ Using liposomes containing immune booster cytokines can increase T cell antitumor function in the TME. According to the lessons learned from the fourth‐generation CAR‐T cells, it may be possible to design engineered NRTs for expressing cytokines and immune‐stimulating growth factors to enhance the effectiveness of treatment and overcome immunosuppressive TME.^[^ ^73^ ^]^ Nevertheless, applying these modifications and using genetic engineering methods increases the cost and complexity of treatment.

## 4 NRTs in Cancer Immunotherapy

Genetically modified T cells or adoptive T cell therapy is being studied as a potential therapeutic approach in treating several human cancers. Researchers frequently use chimeric antigen receptor (CAR) T cells, MHC class I‐restricted T cell receptor (TCR), CD8^+^, CD4^+^, and bulk T cells to treat patients with cancer.^[^ ^74^ ^]^ In this section, the outcomes of some of these studies are discussed. KRAS is one of the human cancers' most common mutant proto‐oncogenes, occurring in the single amino acid at codon 12.^[^ ^75^, ^76^ ^]^ Based on HLA‐peptide prediction algorithms, HLA‐A * 11: 01 can effectively deliver antigens derived from KRAS mutated variants to specific T cells. Animal studies show that TCRs isolated from the studied animals could actively identify mutated G12V and G12D KRAS variants. Peripheral blood lymphocytes transduced with these TCRs can also detect mutated KRAS neoantigens in HLA‐A * 11: 01^+^ tumor cell lines and reduce tumor growth in xenograft cancer models.^[^ ^77^ ^]^ Identifying tumor‐specific neoantigen encoded by mutant genes is limited to resident and infiltrated CD8^+^ T cells in tumor tissue. There is still no precise information about what happens to circulatory CD8^+^ T cells.^[^ ^54^, ^78^ ^]^ A study showed that the intratumoral expression of PD‐1 could be useful as a biomarker in identifying resident neoantigens‐specific CD8^+^ T cells in tumor tissue.^[^ ^79^ ^]^ This study showed that despite the low frequency of CD8+ PD‐1+ cells, these cells could identify neoantigens in the circulation of patients with melanoma cancer. Also, neoantigen‐specific CD8^+^T cells and gene‐engineered lymphocytes expressing neoantigen‐specific TCRs isolated from the peripheral blood of these patients could detect autologous tumors. As a result, PD‐1 can be used to identify various types of specific anti‐tumor CD8^+^ T cells and develop personalized medicine‐based treatment approaches such as NRT immunotherapy.^[^ ^80^ ^]^ It has been demonstrated that microwell culturing can prevent the overgrowth of non‐reactive T cells. One study showed that PD‐1^+^ T cells could effectively identify neoantigen‐reactive TCRs. For example, with this approach, researchers identified three HLA II‐restricted TCRs targeting the TP53^G245S^ hot‐spot mutation in a patient with ovarian cancer and one HLA II‐restricted KRAS^G12V^‐reactive TCR in another patient. Therefore, this method could be a highly sensitive platform to separate clinically applicable NRTs or their TCRs for personalized immunotherapy of cancers.^[^ ^81^ ^]^ In a study on melanoma, it has been described that a new screening method employing mining WES data to recognize mutated proteins expressed by tumor cells in patients with cancer. Neoantigens were identified using an HLA‐binding algorithm along with antigens recognition by TILs. In addition, candidate mutated T cell epitopes were synthesized and evaluated, and NRTs recognized neoantigens expressed on the surface of autologous tumor cells in patients with melanoma. The outcomes showed that this approach could reduce tumor progression. Another advantage of this method is identifying neoantigens recognized by T cells without screening for cDNA libraries from the tumor, which is complex and laborious.^[^ ^54^ ^]^ Recently, an investigation reported that high‐immunogen neoantigens could be screened from patients with non‐small‐cell lung carcinoma (NSCLC) through the WES of patient specimens and machine‐learning‐based neoantigen predictions. Furthermore, NRTs showed effective antitumor responses in tumor‐bearing mouse models and transgenic mice. These findings indicated that developing neoantigen‐based personalized immunotherapies could efficiently treat NSCLC.^[^ ^82^ ^]^ Other clinical trials have been designed on various human cancers and the use of neoantigen‐specific T cells that are still recruiting patients, and their results will be acknowledged in the future. The most important of these clinical trials are NCT03354390 (neoantigen: HERV‐E), NCT04146298 (neoantigen: KRAS^G12V^), NCT03745326 (neoantigen: KRAS^G12D^), and NCT03412877 (neoantigen: individualized).^[^ ^36^ ^]^

## 5 NRT Immunotherapy in Colorectal Cancer

Despite the wide variety of immunotherapy‐based approaches, most of these methods, such as checkpoint blockers, have not successfully treated CRC.^[^ ^14^ ^]^ Accordingly, some rare studies explored the practicability and usefulness of neoantigens‐based immunotherapy in treating CRC.^[^ ^83^, ^84^, ^85^ ^]^ A study of CRC subsets showed the importance of mutations in genes that are involved in DNA repairs, such as *MLH1*, *MSH2*, *MSH6*, *POLE*, *EXO1*, and *MUTYH*, because these alterations can lead to the formation of neoantigens and disruption of antigen presentation to effector T cells in CRC. Therefore, recognizing neoantigens can be useful in cancer immunotherapy.^[^ ^86^ ^]^

Evidence demonstrated that in patients with inflammatory bowel disease (IBD), the colon mucosa contains many immune cells; therefore, detection and response to epithelial cells expressing neoantigen by the immune system is enhanced. Comparative analysis of neoantigen burdens in sporadically arising CRCs and ulcerative colitis‐associated CRCs employing multi‐region whole‐exome and whole‐genome sequencing data (for neoantigen prediction) and NeoPredPipe (for evaluation of immunogenic neoantigens diversity), showed that the neoantigen burden of sporadically arising CRCs was higher than colitis‐associated CRCs. While colitis‐associated CRCs have a more grade of intra‐tumor heterogeneity because by excluding CRCs with microsatellite instability, colitis‐associated CRCs had relatively higher numbers of sub‐clonal neoantigens per clonal neoantigens.^[^ ^87^ ^]^

**Figure 1:**
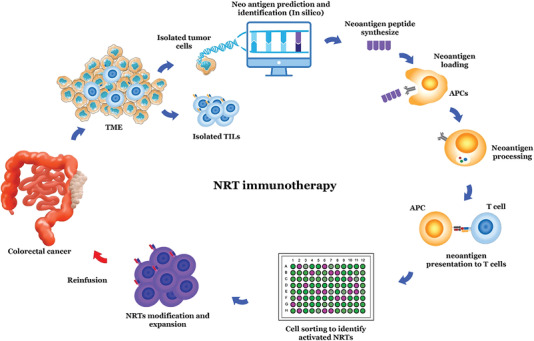
NRT immunotherapy procedure for patients with CRC. In CRC, after invasive sampling, mutated tumor cells, normal cells as well as CD8+TILs are isolated, and neoantigens are determined by sequencing and in silico methods and comparison of mutated tumor cells and normal cells. According to the neoantigen sequence, the desired peptides are synthesized. These peptides are then identified and captured by antigen‐presenting cells and presented to isolated TILs. Next, the responder and activated T cells are identified and returned to the patient's body following optimization. After entering the patient's body, NRT cells identify the neoantigens expressed and presented by tumor cells, and NRTs eventually kill these cells.

As discussed before, KRAS, a commonly mutated proto‐oncogene, could be detected in several human malignancies.^[^ ^88^ ^]^ Previous studies showed that KRAS variants such as G12D and G12V are present in 20% to 30% of CRCs.^[^ ^77^, ^89^, ^90^, ^91^, ^92^, ^93^ ^]^ An investigation has shown that HLA‐C*08:02‐restricted TIL‐based immunotherapy targeting KRAS^G12D^ oncogene mutation could reduce tumor development in metastatic CRC.^[^ ^18^ ^]^ Metastasis to lung regressed following infusion of 1.11 × 10^11^ HLA‐C*08:02–restricted KRAS^G12D^ targeting TILs. Accordingly, the infusion of CD8^+^ T cells targeting mutated KRAS variants could be effective anti‐tumor effects in human metastatic malignancies that expressed HLA‐C*08:02 and KRAS^G12D^, including CRC.^[^ ^18^, ^94^ ^]^ As mentioned, infiltrated neoantigen‐specific CD8^+^ T cells in the TME can inhibit tumor growth. However, detecting these cells in the peripheral blood of patients with metastatic cancers is not yet fully understood. In CRC, it has been reported that using a highly sensitive in vitro stimulation method and specific CD8^+^ peripheral blood memory T cells empowerment targeting the SMAD5 and mucin 4 (MUC4) neoantigens could be effective in the development of personalized NRT‐based immunotherapy.^[^ ^95^ ^]^ Due to identifying recurrent neoantigen in CRC, a study used WES on 1779 data obtained from seven published CRC cohort studies. In this study, common HLA genotypes were employed to predict neoantigens. According to the WES data, mutations were 8% for KRAS^G12D^, 5.8% for KRAS^G12V^, 3.5% for PIK3CA ^E545K^, 2.8% for BMPR2 ^N583Tfs44^, and 2.5% for PIK3CA ^H1047R^.^[^ ^50^ ^]^ Although this study was performed on CRC, the mentioned mutations have been identified in several human metastatic malignancies that can be targeted in immunotherapy‐based therapeutic approaches. In consensus, molecular subtypes (CMS) of MMR‐d CRC patients, specific neoantigen immune responses were realized against NIC3, NIC4, and NIC15 mutant peptides. In the mentioned patients, NRTs (CD8^+^CD39^+^CD103^+^T cell subset) could be detected in the tumor milieu, where the TGF‐β level is significantly elevated to suppress anti‐tumor immune responses, and this problem can be partially alleviated with combination therapies.^[^ ^96^ ^]^ Another study disclosed the presence of NRTs in several types of MMR‐p‐associated metastatic gastrointestinal malignancies, such as CRC. The High‐throughput immunologic mutant gene product screening identified 123 unique NRTs from 83% of patients with frequent gastrointestinal cancers. T‐cell identification assays also approved the somatic nonsynonymous encoded mutations' immunogenicity and their product.^[^ ^97^ ^]^ These findings suggest that in most primary and metastatic stages of epithelial cancers, such as CRC, peptides derived from mutated genes can be detected by neoantigen‐specific T cells and induce an anti‐tumor response against them which is considered the foundation of NRT immunotherapy.^[^ ^97^ ^]^ Due to the NRTs induction, an investigation employed HLA‐A2.1‐restricted neoantigens in HLA‐A2.1/ Kb transgenic mice, and mouse‐derived lymphocytes were transferred into the transgenic human CRC tumor‐bearing C57BL/6nu/nu mice. The findings showed that the induced tumor in the xenograft CRC mice model was regressed significantly. Therefore, these data indicated that induction of NRTs by neoantigens could be a potential tactic in treating CRC (**Figure** **1**).^[^ ^12^ ^]^ An investigation showed that the neoantigens from several patients with CRC, including TSHZ3‐L523P, RARA‐R83H, TP53‐R248W, EYA2‐V333I, NRAS‐G12D, TASP1‐P161L, RAP1GAP‐S215R, MOSPD1‐V63I, NAV2‐D1973N, HAVCR2‐F39V, SEC11A‐R11L, SMPDL3B‐T452M, LRFN3‐R118Q, and ULK1‐S248L stimulated a potent NRT response than in peripheral blood lymphocytes obtained from CRC patients. Additionally, the adoptive transfer of mutant peptides‐activated NRTs could effectively suppress tumor growth in mice models. Accordingly, neoantigen‐containing immunogenic peptides might be potential candidates for peptide‐based personalized immunotherapy.^[^ ^19^ ^]^ Analyzing synthetic peptide‐activated TILs isolated from MMR‐proficient CRCs showed that neoantigen‐directed reactivity was only encountered in bulk TIL populations which could be attributed to CD4^+^ T cells and in CD103^+^CD39^+^ CD8^+^ T cell subsets. This reactivity was not detected in single‐positive or double‐negative subsets. Thus, CD39/CD103 coexpression can be considered a hallmark of neoantigen‐specific CD8^+^ T cells in MMR‐proficient CRCs, facilitating engineered T cell receptor therapies or adoptive T cell transfer.^[^ ^20^ ^]^

Recently, a study showed that stimulation of peripheral blood T cells and TILs by cryptic and neoantigenic peptides originating from single nucleotide substitutions in *CTNNB1, TRIT1*, and *IQGAP1* genes amplified the release of cytotoxic granules in the presence of autologous cancer cells, improving the recognition of these cells and antitumor responses. Moreover, following tetramer staining, it was revealed that most TILs and peripheral blood T cells were specific for IQGAP1 neoantigen.^[^ ^98^ ^]^

Regarding the low tumor mutational burden in some tumors, the abundance of neoantigens in them is limited. An alternative chemical‐based compound (RECTAS) to generate splice‐neoantigens in CRC mice models. Findings demonstrated that activated CD8^+^ T cells by RECTAS‐induced neoantigens could significantly inhibit tumor growth and development. Additionally, this approach improved the effectiveness of immune checkpoint inhibitors.^[^ ^99^ ^]^ Another investigation on 196 screened peptides reported that stimulation of enriched neoantigen‐specific TILs, specially HDHD3, could increase the expression of CD137 (4‐1BB), a member of the tumor necrosis factor (TNF) receptor superfamily T cell costimulatory receptor, CD134, (OX40), TNFα, IFNγ, and granzyme B than in non‐reactive TILs.^[^ ^100^ ^]^ Therefore, screening and identifying specific neoantigens can be effective in more targeted treatment of CRC and improving the NRT‐mediated antitumor responses.

## 6 Challenges and Opportunities in NRT Immunotherapy

Due to the high frequency, specificity, and consistency of the neoantigens, cancer therapy using neoantigen‐based cancer vaccines and NRTs is considered an attractive therapeutic tactic.^[^ ^77^ ^]^ However, because of the infancy of these methods, their development rate has not been speedy.

Methods such as WES, which are performed at the genomic scale, are commonly used to identify neoantigens, but the emergence of other new methods, such as deep learning and multi‐omics, can also be beneficial in recognizing more neoantigens.^[^ ^101^ ^]^ In the case of NRT immunotherapy, despite this method's benefits, there is still a long way to go before it can be optimized for cancer treatment. Moreover, researchers face two significant challenges to achieving the long‐term and successful anti‐tumor effects of NRTs: rejuvenating existing cells and differentiating immune stem cells to target cells. Following adoptive cell therapies, the fate of engineered T cells is not yet fully understood. Studies based on the analysis of lymphocyte‐associated genes after one month of infusion showed that the expression of stimulus and activating molecules of T cells was significantly downregulated, while inhibitory molecules such as PD‐1 were upregulated.^[^ ^102^ ^]^ Identification of neoantigens, expansion of NRTs, immunosuppressive TME, anti‐inflammatory mediators, expression of inhibitory molecules such as PDL‐1 and CD160 by tumor cells, ineffective trafficking NRTs into the TME, and the high cost of personalized medicine are known as other important limitation of NRT immunotherapy.^[^ ^103^ ^]^ Examination of tumor tissues nine months after NRT immunotherapy showed that tumor cells used an interesting strategy to escape from NRTs. The disappearance of the chromosome 6 haplotype encoding the HLA‐C*08: 02 molecules in tumor cells leads to the downregulation of MHCs, resulting in an escape from NRTs recognition. Therefore, MHC restriction in NRT immunotherapy and TCR‐T therapy is one of the most critical challenges in cancer therapy.^[^ ^13^ ^]^

Along with the complexities of *in silico* methods in identifying neoantigens, the low immunogenicity of the predicted candidates for neoantigens is another problem. Advanced tools such as NetMHCIIpan and NetMHCpan that rely primarily on the binding affinity between peptide and HLA molecules with a low validity rate of ≈20 to 30 percent led to the use of MuPeXI as a computational tool that combines integrates RNA expression, mutant allele frequency, similarity to self‐peptides, and binding affinity to realize the neoantigen candidate's possibility in anti‐tumor immune responses.^[^ ^104^ ^]^ The heterogeneity of tumors is another obstacle in cancer immunotherapy.^[^ ^105^ ^]^ It has been revealed that there is a heterogeneous tumor antigen pool while the infused NRTs are designed to recognize a single neoantigen.^[^ ^106^ ^]^ In the NRTs preparation phase, the selection bias of the neoantigens sequencing and the relative coverage of TCRs in the execution phase led to a decrease in the effectiveness of anti‐tumor functions.^[^ ^36^ ^]^ Mechanisms of resistance of tumor cells, especially the escape of these cells from NRTs by reducing the expression of MHC molecules and reducing or changing tumor antigens through uninterrupted mutations subsequently of the treatment period, are other factors in the failure of NRT immunotherapy. Therefore, using other cancer treatment methods such as surgery, chemotherapy, radiation therapy, cell therapy, cytokine therapy, and immune checkpoint blockers with NRTs may strengthen anti‐tumor responses and overcome tumor escape mechanisms.^[^ ^107^, ^108^ ^]^ NRT immunotherapy is a time and cost‐consuming method.

Moreover, limited autologous lymphocytes are usually used to prepare NRTs. Using “off the shelf” T cells have been considered to solve this problem because this strategy can effectively unify products at a low cost quickly.^[^ ^39^, ^40^, ^109^ ^]^ Employing cancer vaccines to improve cell‐mediated immunity is considered one of the novel immunotherapy opportunities for cancer therapy. Compared to NRT immunotherapy, cancer vaccines such as mRNA‐based neoantigen vaccines have advantages, including low manufacturing costs, stability, scalability, reliability, efficacy, and fast manufacturing.^[^ ^110^, ^111^ ^]^

Finally, due to the discovery of tumor antigens in exosomes released by tumor cells and misfolded proteins and nucleic acids derived from these cells, it may be possible to omit invasive sampling methods such as surgery to identify tumor antigens and early diagnosis of cancer.^[^ ^112^ ^]^ Replacement of the liquid biopsy tools by invasive sampling methods can facilitate cell therapy procedures.^[^ ^113^ ^]^

## 7 Concluding Remarks

NRT immunotherapy can be an innovative method to treat human cancers such as CRC. However, due to the involvement of other sciences, such as computers and genetics, different dimensions of this method need further studies to reduce the complexity, cost, and time of NRTs production. Accordingly, the use of combination therapies in the treatment of CRC may be a decent alternative to monotherapy with NRTs, because, according to existing experience and the complexities of the TME, monotherapy cannot cover all the challenges. Developing neoantigen prediction methods, such as TruNeo, which is based on several biological processes rather than only predicting peptide‐MHC binding affinity methods, using novel algorithms, and identifying neoantigens‐specific T cells in the preparation phase, can improve the effectiveness of the NRT immunotherapy in CRC. Moreover, the expansion of NRTs and increasing their persistence to enhance anti‐tumor function in vivo are essential factors in the success of NRT immunotherapy. Although several clinical trials have not been completed yet, their results can help fix this therapeutic approach's bugs soon. On the other hand, developing artificial intelligence and computational methods in predicting immunogenic neoantigens and peptides can boost CD8^+^ T cell‐mediated antitumor immune responses.

## Conflict of Interest

The authors declare no conflict of interest.

## Author Contributions

G.‐L.C. and D.‐X.K. contributed equally to this work. Both G.L.C. and D.X.K. equally contributed to writing the manuscript and sourcing references for the review. Y.L. conceived the outline of this paper and participated in critical review and further revision of the manuscript. All authors contributed to critical discussions and finalizing the manuscript before submission. They have all given approval to the final form of the manuscript.
